# Mucinous adenocarcinoma in the axilla of undetermined origin

**DOI:** 10.1093/jscr/rjaf258

**Published:** 2025-05-14

**Authors:** Chloe Spillane, Roisin Daly, Mar Cotter, Kristali Ylli, Wala Eljack, Ciaran Sheehan, Corina Girleanu, Michael Bennett, Aongus Twomey

**Affiliations:** Department of Surgery, Mercy University Hospital, Grenville Place, Cork, Ireland, T12 WE28; Department of Surgery, Mercy University Hospital, Grenville Place, Cork, Ireland, T12 WE28; Department of Surgery, Mercy University Hospital, Grenville Place, Cork, Ireland, T12 WE28; Department of Surgery, Mercy University Hospital, Grenville Place, Cork, Ireland, T12 WE28; Department of Surgery, Mercy University Hospital, Grenville Place, Cork, Ireland, T12 WE28; Department of Surgery, Mercy University Hospital, Grenville Place, Cork, Ireland, T12 WE28; Department of Pathology, Cork University Hospital, Wilton, Cork, Ireland, T12 DFK4; Department of Pathology, Cork University Hospital, Wilton, Cork, Ireland, T12 DFK4; Department of Surgery, Mercy University Hospital, Grenville Place, Cork, Ireland, T12 WE28

**Keywords:** primary of unknown origin in the axilla, mucinous adenocarcinoma, primary cutaneous mucinous adenocarcinoma, mucinous adenocarcinoma of the axilla

## Abstract

Mucinous adenocarcinoma (MAC), accounts for ⁓1% of all cancer diagnoses. We present a case of a 75-year-old male who, after presenting for an elective inguinal hernia repair, disclosed concerns for a new lesion in his right axilla which was removed at the same time. Investigations revealed a MAC of unclear origin, with immunohistochemistry suggesting either a breast or primary cutaneous mucinous adenocarcinoma (PCMC). The patient was asymptomatic with an otherwise normal examination. Extensive investigations failed to identify any primary source. Axillary node clearance was performed, resulting in 0/9 positive nodes. After multi-disciplinary team discussion, this patient (who remains asymptomatic) will be kept under close clinical surveillance, with yearly computerized tomography scans. To our knowledge, minimal presentations have been reported in the literature. Thus, our case report is a unique addition of an atypical presentation of PCMC in the axilla.

## Introduction

Mucinous adenocarcinoma (MAC) are malignant tumors in which ˃50% of the tumor is comprised of extracellular mucin, with overt malignant epithelial cells present in clumps, layers, or as individual cells [1]. MAC is a rare malignancy, accounting for 1% of all cancer diagnoses [2]. Outside of its occurrence in more common primary sites, such as colorectal and breast cancers, our knowledge of MAC depends largely on case reports. This is particularly true of primary cutaneous mucinous adenocarcinoma (PCMC) which, according to Breiting *et al.*, has an incidence of 0.07/million person-years [3].

## Case report

**Figure 1:**
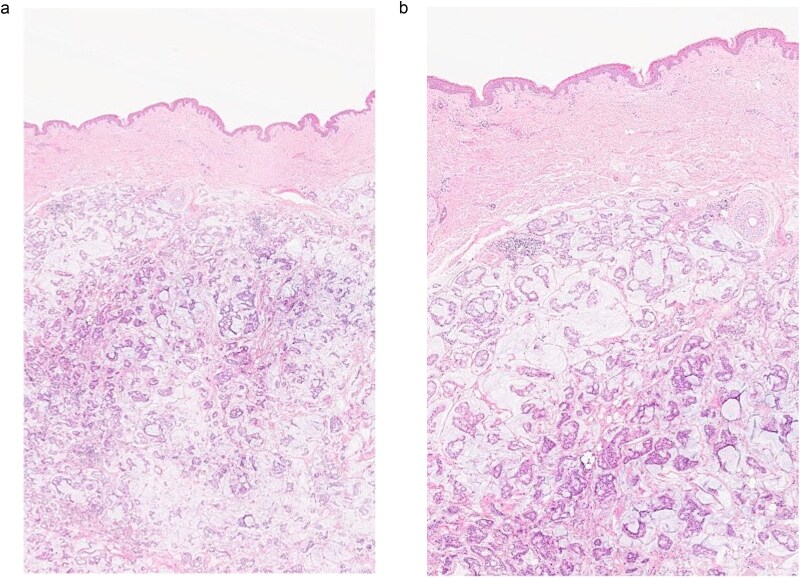
(a, b) Section of skin showing dermal lesion composed of nests of tumor cells floating in pools of extracellular mucin.

**Figure 2:**
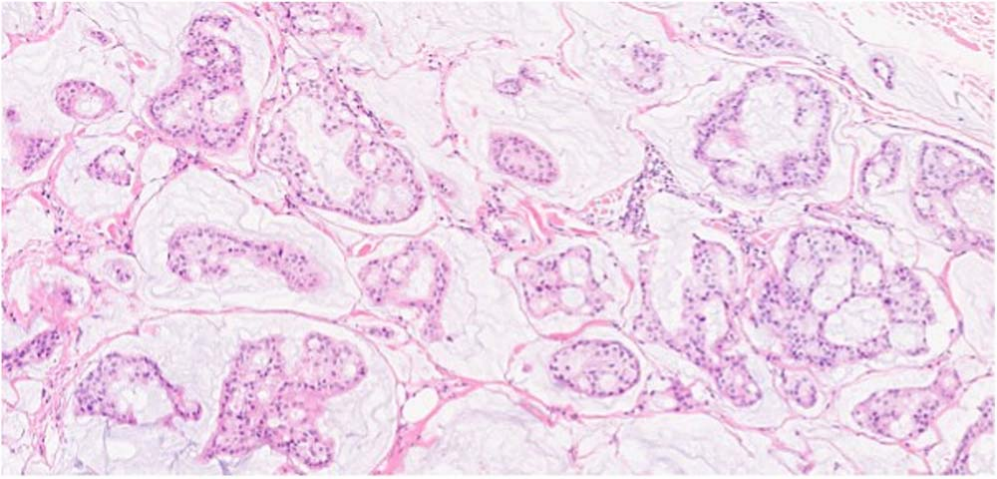
Section showing tumor cells with low to intermediate nuclear grade floating in pools of extracellular mucin separated by fibrous septa.

**Figure 3:**
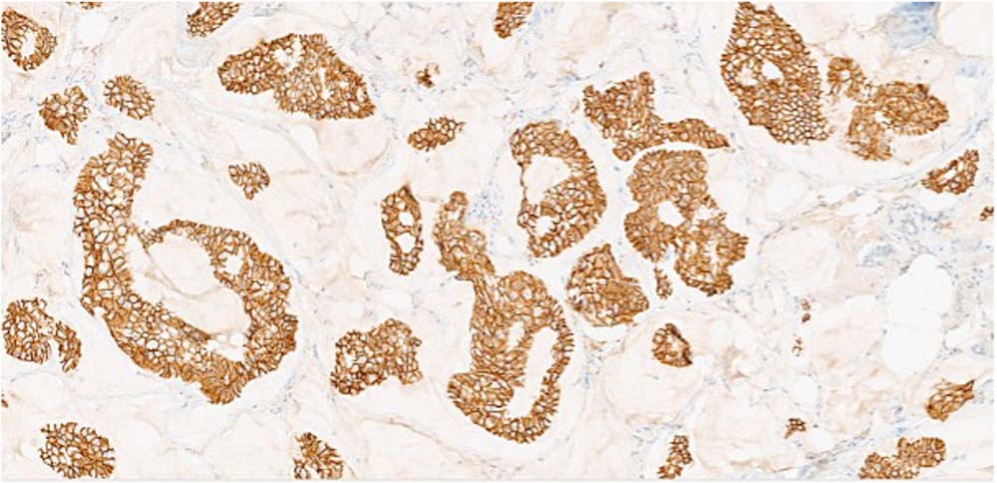
Section of tumor showing HER2 expression in the tumor cells.

**Figure 4:**
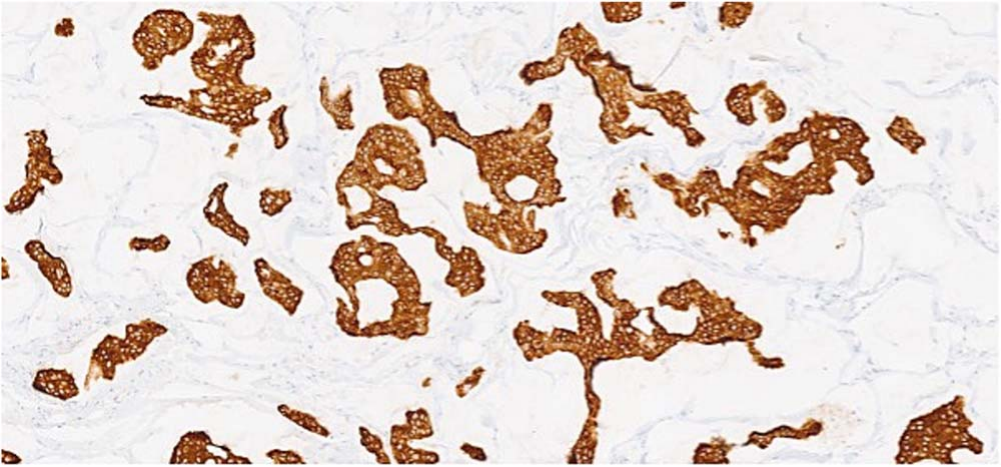
Section of tumor showing strong positivity for CK7.

**Figure 5:**
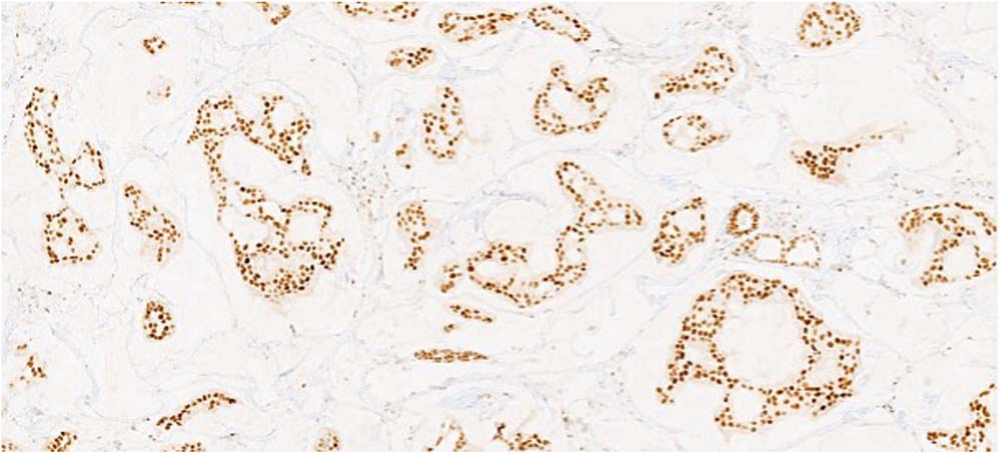
Section of tumor showing strong nuclear positivity.

**Figure 6:**
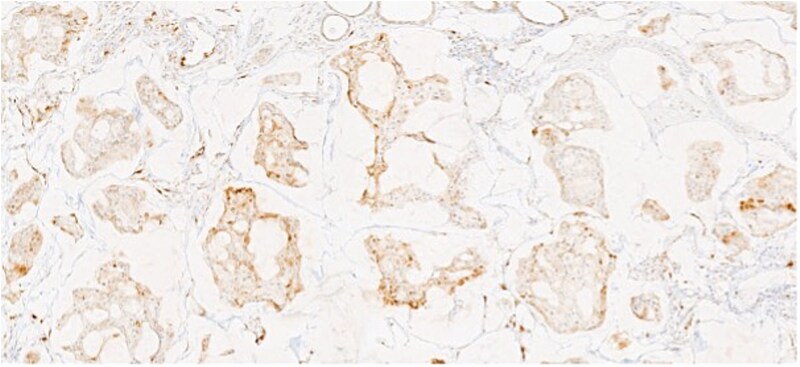
Tumor cells with focally positivity for p16.

Herein, we present a 75-year-old man who, having been referred to our service with an inguinal hernia, raised concern about a palpable mass present in his right axilla. The main differential on initial examination was that of a simple epidermoid cyst, and consent was obtained for its removal during his elective hernia repair. Subsequent histology however, revealed a well differentiated MAC. There was extensive involvement of the deep and superficial reticular dermis (Figs 1and 2). Lymphovascular invasion was present. Sweat glands were focally involved by the carcinoma. The differential diagnosis included PCMC and metastasis from other primary sites of MAC such as breast, lung and gastrointestinal. Immunohistochemical profiling was performed on the specimen. CK 7, HER2 (4B5, 3+), GATA3 (strong, diffuse), and P16 (focal, weak) were positive (Figs 3–6). SOX10, CK 20, CDX2, TTF-1, p40, ER, and PR were negative which supported the diagnosis of MAC with either breast or PCMC origin based particularly on GATA3 expression.

This patient has been asymptomatic. He had no medical co-morbidities of note. No abnormalities were present on breast examination, nor did he have any other abnormal cutaneous lesions or lymphadenopathy. No primary tumor was identified on endoscopic investigation. Imaging in the form of computerized tomography (CT) of the thorax, abdomen and pelvis, as well as subsequent positron emission tomography (PET) also failed to identify an overt primary tumor. Mammography and ultrasound of the breast was normal, as was ultrasound of thyroid. Tumor markers included CEA of 5 ng/ml (range 0-5 ng/ml), CA 19–9 of 19 U/ml (range 0–37 U/ml), and PSA of 6.78 ng/ml (range 0–4.9 ng/ml). Following multi-disciplinary team (MDT) discussion, a right axillary node clearance was recommended.

A level III axillary node clearance was performed, with no acute complications. In total nine lymph nodes were isolated, with no evidence of metastatic carcinoma (0/9). With no definite primary MAC identified, further MDT discussion with oncology input, deemed that chemotherapy would not be of benefit at this point. Radiotherapy of the axilla was also contraindicated, given the axillary clearance. The patient (who remains asymptomatic) was thus commenced on close clinical surveillance with yearly CT imaging.

## Discussion

To our knowledge, no such similar presentations of MAC in the axilla have been reported in the literature. This case highlights two possible differential diagnoses, that of breast MAC or PCMC. PCMC is the most likely diagnosis, as it is determined by exclusion following a comprehensive evaluation and investigations as discussed. Histologically, PCMC is indistinguishable from metastatic carcinoma of the breast, gastrointestinal tract or lung. However, several stains, such as p63, CK5/6, and D2–40, have been reported to aid in differentiating primary from metastatic MAC of the skin [4]. It is essential to conduct a metastatic work-up, which must yield negative results to confirm that the lesion is not a metastasis from another primary site and to establish a definitive diagnosis of PCMC [5].

PCMC is a rare low-grade malignant neoplasm derived from eccrine origin which was first described by Lennox *et al.*[6]. It generally follows a slow, indolent clinical course, often remaining undiagnosed for several years. Clinically, it is frequently misdiagnosed as an epidermal cyst, sebaceous carcinoma, cystic basal cell carcinoma, squamous cell carcinoma, neuroma, or pilomatrixoma [5]. The head and neck, particularly around the eyelids, are the most common sites. Other locations include the scalp (17%), face (14%), axilla (9%), chest or abdomen (7%), vulva (4%), extremities (2%), neck (2%), canthus (2%), groin (1%), and ear (1%) [7, 8]. They typically range in size from 1 to 8 cm, with an average diameter of 1.8 cm before excision, although larger cases have been documented in the literature. The nodules are well-defined and unencapsulated, often firmly attached to the dermis, making them difficult to “shell out” [9].

As with many rare nonmelanoma tumors, there is no established standard of care for the surgical management of PCMC. However, the preferred treatment is wide surgical excision, including regional lymph node dissection. When wide surgical excision is not feasible, Mohs surgery may be considered as an alternative. Mohs surgery is recommended as it offers complete margin control while preserving unaffected tissues, leading to favourable cosmetic outcomes [10]. Its use has been shown to achieve higher rates of disease control and improved 5-year overall survival [11, 12]. PCMC does not respond to radiation therapy or chemotherapy. Local recurrence occurs frequently (20%–30%), while distant metastases are less frequent (9.6%). These tumors more commonly invade surrounding tissues through direct extension, often due to satellite clusters of tumor cells surrounding the primary nodule, as well as through regional lymph node involvement. Fatal cases of PCMC are exceedingly rare, with fewer than five reported instances, primarily linked to multiple recurrences and extensive metastatic disease [10]. In the absence of standardized treatment guidelines for PCMC, patients are generally advised to undergo close postoperative surveillance. Monitoring levels of CEA and CA15.3 can aid in the early detection of recurrence or metastatic disease, however this is only highlighted in one case report [13].

In summary, MAC of undetermined origin remains a challenge for clinicians and requires MDT input to help guide management. This case emphasizes the necessity of a standardized approach in investigating MAC, the importance of immunohistochemistry in narrowing down potential diagnoses, and the overall paucity of literature on this subject matter.
